# Incorporating the metabolic ratio (C-_VRC_/C-_VNO_) into a LASSO-logistic model for predicting voriconazole-induced DILI: development and web-based implementation

**DOI:** 10.3389/fphar.2026.1857552

**Published:** 2026-07-06

**Authors:** Jing Ling, Xueling Fu, Xuping Yang, Lulu Dong, Yan Jiang, Nan Hu

**Affiliations:** 1 Department of Pharmacy, The First People’s Hospital of Changzhou/The Third Affiliated Hospital of Soochow University, Changzhou, China; 2 School of Basic Medicine and Clinical Pharmacy, China Pharmaceutical University, Nanjing, China

**Keywords:** drug-induced liver injury, lasso-logistic regression, nomogram, prediction model, risk factors, voriconazole

## Abstract

**Objective:**

Voriconazole (VRC) is widely used as an antifungal agent, yet its potential to cause drug-induced liver injury (DILI) remains a major clinical concern. Therefore, this study aims to identify the relevant risk factors for VRC-induced DILI, construct a predictive model using LASSO-Logistic regression, and present it as a nomogram. Additionally, a web-based prediction tool was developed to facilitate clinical application, ultimately providing a scientific basis for early risk assessment of DILI in patients receiving VRC therapy.

**Methods:**

Clinical data of inpatients who received VRC for the treatment or prophylaxis of fungal infections at the First People’s Hospital of Changzhou from March 2022 to June 2025 were collected retrospectively. The patients were divided into a DILI group and a non-DILI group according to the diagnostic criteria for DILI. Univariate analysis was used to screen for variables with intergroup differences, followed by LASSO regression for further feature optimization. The screened variables were incorporated into a multivariate Logistic regression to construct a nomogram prediction model for DILI. The receiver operating characteristic (ROC) curve, calibration curve and decision curve analysis (DCA) were adopted to evaluate the discrimination, calibration and clinical applicability of the model, with the Bootstrap method for internal validation. Additionally, a web-based interactive prediction calculator was developed based on R Shiny.

**Results:**

A total of 250 patients were enrolled, among whom 47 developed DILI. Univariate analysis revealed statistically significant differences between the DILI group and the non-DILI group in seven variables, namely renal replacement therapy, concomitant bacterial infection, steady-state trough concentration of VRC (C-_VRC_), C-_VRC_/daily dose (C-_VRC_/DD), the ratio of C-_VRC_ to VRC N-oxide concentration (C-_VRC_/C-_VNO_, MR), albumin (ALB) and serum creatinine (Scr) (all *P* < 0.05). LASSO regression further identified five variables associated with VRC-induced DILI: concomitant bacterial infection, C-_VRC_, MR, ALB and Scr. Multivariate Logistic regression analysis demonstrated that C-_VRC_, MR, ALB and Scr were independent risk factors for VRC-induced DILI (all *P* < 0.05). A nomogram prediction model was constructed based on the above factors, with the area under the ROC curve (AUC) of 0.818 (95% confidence interval [CI]: 0.748–0.888). The calibration curve showed a good fit between the predicted values and actual observed values of the model. DCA indicated that the model yielded favorable clinical net benefits within a certain range of threshold probabilities, and the Bootstrap method verified the good stability of the model. The developed web-based calculator enables the calculation of DILI risk probability in individual patients.

**Conclusion:**

C-_VRC_, MR, ALB and Scr are important predictors of VRC-induced DILI. The nomogram prediction model constructed based on LASSO-logistic regression has good predictive performance and clinical applicability, and the developed web-based interactive prediction calculator improves the clinical convenience of the model. This model can be effectively used for the early risk assessment of DILI in patients receiving VRC, providing support for clinical individualized medication and risk prevention and control.

## Introduction

Voriconazole (VRC) is a second-generation, broad-spectrum triazole antifungal agent that exerts potent antifungal effects by disrupting fungal cell membrane integrity and function. It exhibits high activity against *Aspergillus*, *Candida* and *Fusarium*. Current clinical guidelines recommend VRC as a first-line agent for the treatment or prevention of invasive aspergillosis, candidemia in non-neutropenic patients, and other fungal infections (Patterson et al., 2016). Despite its well-established efficacy, VRC demonstrates substantial interindividual pharmacokinetic variability in clinical practice and has a narrow therapeutic window. It is frequently associated with adverse reactions, among which drug-induced liver injury (DILI) is particularly common and clinically significant (Eiden et al., 2007; Tang et al., 2021). A real-world study based on the FDA Adverse Event Reporting System database indicated that VRC is one of the leading antifungal agents associated with DILI, accounting for up to 32.45% of reported case (Zhou et al., 2022). Moreover, VRC had a significantly higher incidence of abnormal liver function tests than fluconazole and echinocandins (Rakhshan et al., 2023). Furthermore, VRC-induced liver injury encompasses various pathological patterns, including hepatocellular, cholestatic, and mixed types. Its clinical manifestations range from mild elevation of transaminases to severe cholestasis, and even acute liver failure, which severely affects the treatment course of underlying diseases and patient prognosis, posing a major challenge to its clinical application (Lou et al., 2025). Therefore, identifying high-risk patients for VRC-induced DILI and implementing early interventions to reduce its incidence has become a critical issue that urgently needs to be addressed in clinical practice.

In recent years, increasing efforts have been made to identify risk factors for VRC-induced liver injury. Previous studies have revealed that CYP2C19 gene polymorphisms, plasma concentration, duration of treatment, route of administration, underlying diseases, concomitant medications, and transplant status are among the independent risk factors (Lou et al., 2025; Ren et al., 2025; Wang et al., 2016; Zhang et al., 2021). In particular, the relationship between VRC exposure levels and liver injury has attracted considerable attention. Studies have shown that higher plasma concentrations significantly increase the risk of abnormal liver function tests (Matsumoto et al., 2009). The mechanism underlying VRC-induced liver injury has not yet been fully elucidated. A recent study using quantitative systems toxicology modeling suggested that oxidative stress induced by VRC and its primary metabolite voriconazole N-oxide (VNO) is one of the key mechanisms of hepatocellular injury. VNO contributes to mitochondrial dysfunction by promoting the accumulation of reactive oxygen species and inhibiting antioxidant enzymes (Du Q et al., 2025). This finding indicates that monitoring VRC concentration alone may be insufficient to accurately predict DILI risk, whereas comprehensive monitoring of voriconazole concentration (C_-VRC_), voriconazole N-oxide concentration (C_-VNO_), or the metabolic ratio (C_-VRC_/C_-VNO_, MR) may have important clinical value in risk prediction.

Based on the various risk factors for VRC-induced DILI described above, studies on the development and validation of liver injury prediction models have been gradually conducted by researchers in recent years (Du Q et al., 2025; Feng et al., 2025; Tong et al., 2024; Xiao et al., 2023). These models aim to identify the risk of liver injury by integrating relevant predictors, thereby providing a scientific basis for individualized medication and risk mitigation in clinical practice. Nevertheless, several limitations remain. First, feature selection is often unsystematic, as many studies rely on traditional univariate screening methods, which may lead to overfitting or the omission of important predictors. Second, the set of predictive variables remains incomplete, with most models limited to VRC plasma concentrations, basic biochemical markers, and common inflammatory indicators, while genetic factors such as CYP2C19 gene polymorphisms and metabolite markers like VNO are not adequately integrated. Third, there is a lack of user-friendly clinical tools, making it difficult for clinicians to quickly and conveniently apply these models for risk assessment. Accordingly, this study will utilize retrospective clinical data to integrate multiple variables, including demographic characteristics, clinical conditions, CYP2C19 gene polymorphisms, concomitant medications, C_-VRC_, C_-VNO_, and MR. Key predictors will be identified using LASSO regression and multivariate logistic regression. A personalized risk prediction model for VRC-induced DILI will then be constructed and implemented as a web-based visualization tool to facilitate early identification of high-risk patients and support safer and more precise clinical use of VRC.

## Materials and methods

### Patients

In this single-center, retrospective study, hospitalized patients who received VRC for the treatment or prevention of fungal infections at the First People’s Hospital of Changzhou between March 2022 and June 2025 were enrolled. The inclusion criteria were as follows: (1) age ≥ 18 years; (2) duration of VRC treatment > 5 days with therapeutic drug monitoring (TDM) available; (3) liver function tests performed within 5 days prior to VRC initiation. The exclusion criteria included: (1) concomitant hepatobiliary diseases, including various viral hepatitis, alcoholic liver disease, autoimmune liver disease, or cholangitis; (2) abnormal baseline liver function indicators; (3) absence of liver function tests during VRC treatment; (4) changes in liver biochemical indicators attributed to other causes (e.g., other diseases or medications); (5) non-steady-state trough concentrations; (6) missing clinical data. This study was approved by the Ethics Committee of the First People’s Hospital of Changzhou (Approval No (2023) −038).

Data extracted from the hospital’s electronic medical record system included sex, age, body weight, smoking history, alcohol consumption history, underlying diseases (hypertension, diabetes mellitus, chronic obstructive pulmonary disease, hematological malignancies), type of fungal pathogen, concomitant bacterial infection, use of renal replacement therapy, concomitant medications (methylprednisolone, dexamethasone, proton pump inhibitors, and antibacterial agents), voriconazole dosing (loading dose, maintenance dose), route of administration, treatment duration, and laboratory parameters (alanine aminotransferase, aspartate aminotransferase, alkaline phosphatase, gamma-glutamyl transferase, total bilirubin, albumin, C-reactive protein, and serum creatinine). All baseline laboratory parameters were collected within 48 h prior to the first dose of voriconazole administration and served as baseline predictors for DILI risk assessment.

### C_-VRC_ and C_-VNO_ Determination

**FIGURE 1:**
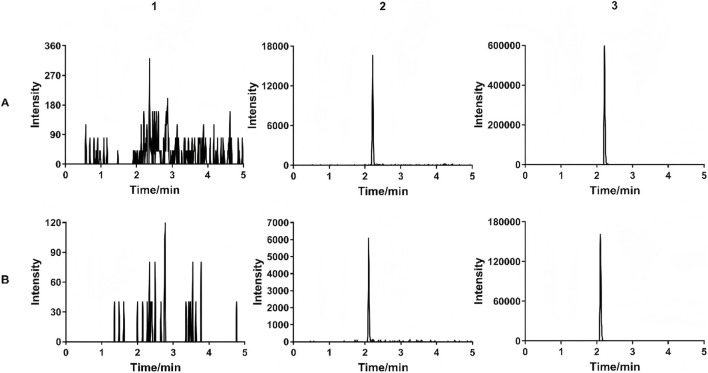
Chromatograms of two compounds **(A)** VRC; **(B)** VNO; 1-blank plasma; 2-blank plasma spiked with standards (LLOQ, VRC: 0.1 μg/mL, VNO: 0.05 μg/mL); 3- blank plasma spiked with standards (Middle quality control, VRC: 3 μg/mL, VNO: 1.3 μg/mL).

For patients receiving a loading dose followed by a maintenance dose for three consecutive days, or for those without a loading dose after five consecutive days, steady-state trough blood samples (2 mL) were collected within 30 min before the next dose. The blood samples were placed in EDTA anticoagulant tubes and centrifuged at 4000 rpm for 5 min. An aliquot of 50 μL plasma was transferred to a 1.5 mL centrifuge tube, mixed with 150 μL of internal standard working solution (250 ng/mL), vortexed for 3 min, and centrifuged at 16,400 rpm for 10 min. Then, 20 μL of the supernatant was transferred to another centrifuge tube and diluted with 180 μL of 50% methanol-water solution. The final solution was placed in an autosampler vial, and C_-VRC_ and C_-VNO_ were measured using LC-MS/MS. The typical chromatogram is shown in Figure 1. For VRC, the accuracy ranged from 100.5% to 113.1% with precision of 4.5%–10.4%. For VNO, the accuracy ranged from 90.0% to 108.4%, and the precision ranged from 5.5% to 13.9%. Stability tests showed that both analytes were stable after storage at 25.0 °C for 6 h, at 4.0 °C for 48 h, and after three freeze-thaw cycles, with mean deviations of measured concentrations from nominal values all within ±15%.

### CYP2C19 genotyping

Genomic DNA was extracted using a blood genomic DNA extraction kit. DNA concentration and purity (A260/A280 ratio between 1.8 and 2.0) were determined using a NanoDrop 2000 spectrophotometer (Thermo Fisher Scientific), and the DNA products were stored at −20 °C. Polymerase chain reaction amplification was performed using the extracted DNA as template with the following primers: for CYP2C19*2, forward primer ATC​AAA​AGC​AGG​TAT​AAG​TCT​AG and reverse primer CACAAATACRCAAGCAGTCA; for CYP2C19*3, forward primer TTCCAATCAKTTAGCTTCACC and reverse primer AAC​TTT​GCC​ATC​TTT​TCC​AG. The reaction conditions were: initial denaturation at 95 °C for 5 min; followed by 35 cycles of denaturation at 95 °C for 30 s, annealing at 55 °C for 30 s, and extension at 72 °C for 30 s; with a final extension at 72 °C for 10 min. The amplified products were sequenced using a 3730XL DNA Analyzer (ABI, United States of America). Based on the CYP2C19 genotype, patients were classified as poor metabolizers (PMs) carrying two loss-of-function alleles (*2/*2, *2/*3, or *3/*3), intermediate metabolizers (IMs) carrying one loss-of-function allele (*1/*2 or *1/*3), and extensive metabolizers (EMs) carrying no loss-of-function alleles (*1/*1).

### DILI determination

Liver injury was defined according to the 2023 Chinese guidelines for the diagnosis and management of drug-induced liver injury (Mao, 2023). Before VRC administration, normal liver function was confirmed. During treatment, liver injury was defined as alanine aminotransferase ≥ 3 × the upper limit of normal (ULN) or alkaline phosphatase ≥ 2 × ULN.

### Causality assessment of VRC-Induced liver injury

Causality assessment between VRC and liver injury was performed using the Roussel Uclaf Causality Assessment Method (RUCAM) for all included patients (Xiao et al., 2025). A RUCAM score of ≥ 8 indicated that liver injury was highly likely attributable to VRC; a score of 6 to < 8 indicated probable; a score of 3 to < 6 indicated possible; a score of 1 to < 3 indicated unlikely; and a score of ≤ 0 excluded VRC as a cause of liver injury. In this study, a RUCAM score of ≥ 3 was defined as VRC-related DILI.

### Statistical analysis

Statistical analyses were performed using SPSS (version 27.0), R (version 4.4.2), and Python (version 3.9.1). Normality was assessed using the Shapiro–Wilk test. Continuous variables with a normal distribution were expressed as mean ± standard deviation and compared using the independent-samples t-test, whereas non-normally distributed variables were presented as median (interquartile range, IQR) and compared using the Mann–Whitney U test. Categorical variables were expressed as frequencies (percentages) and compared using the chi-square (χ^2^) test. Variables with P < 0.05 in univariate analysis were further selected using least absolute shrinkage and selection operator (LASSO) regression. A multivariate logistic regression model was then constructed, and its discriminatory ability was evaluated using receiver operating characteristic (ROC) curves. Calibration of the model was assessed using calibration curves in conjunction with the Hosmer–Lemeshow test, while clinical utility was evaluated using decision curve analysis (DCA). A web-based prediction calculator was developed using an R Shiny application to facilitate clinical implementation of the model. Internal validation was performed using bootstrap resampling with 1,000 iterations. P-value < 0.05 was considered statistically significant.

## Results

### Patient characteristics

A total of 250 patients were included in this study, of whom 47 met the diagnostic criteria for DILI (score ≥ 3) and were assigned to the DILI group, while the remaining 203 were assigned to the non-DILI group. A comparison of clinical characteristics between the two groups is shown in Table 1. Univariate analysis showed that, compared with the non-DILI group, the DILI group exhibited statistically significant differences in the use of renal replacement therapy, concomitant bacterial infection, C_-VRC_/DD, C_-VRC_, MR, ALB, and Scr (*P* < 0.05). No statistically significant differences were observed between the two groups for the remaining variables (*P* > 0.05).

**TABLE 1:** Comparison of clinical characteristics in patients with and without voriconazole-Induced DILI.

Clinical data	Non-DILI group (*n* = 203)	DILI group (*n* = 47)	χ^2^/Z	*P*
Gender [*n* (%)]	​	​	χ^2^ = 0.421	0.516
Female	57 (28.1%)	11 (23.4%)	​	​
Male	146 (71.9%)	36 (76.6%)	​	​
Age [year, M(P_25_,P_75_)]	72.00 (66.00,78.00)	75.00 (67.00,78.00)	Z = −0.751	0.453
Weight [kg, M(P_25_,P_75_)]	65.00 (56.25,70.00)	60.00 (50.00,70.00)	Z = −1.650	0.099
Smoking [*n* (%)]	61 (30.0%)	14 (29.8%)	χ^2^ = 0.001	0.972
Drinking [*n* (%)]	26 (12.8%)	8 (17.0%)	χ^2^ = 0.577	0.447
Renal replacement therapy [*n* (%)]	20 (9.9%)	11 (23.4%)	χ^2^ = 6.453	0.011
CYP2C19 genotype [*n* (%)]	​	​	χ^2^ = 2.806	0.246
EMs	85 (41.9%)	15 (31.9%)	​	​
IMs	95 (46.8%)	23 (48.9%)	​	​
PMs	23 (11.3%)	9 (19.1%)	​	​
Hypertension [*n* (%)]	115 (56.7%)	24 (51.1%)	χ^2^ = 0.483	0.487
Diabetes [*n* (%)]	55 (27.1%)	9 (19.1%)	χ^2^ = 1.265	0.261
COPD [*n* (%)]	33 (16.3%)	5 (10.6%)	χ^2^ = 0.935	0.334
Hematologic malignancy [*n* (%)]	39 (19.2%)	11 (23.4%)	χ^2^ = 0.419	0.517
Pathogen [*n* (%)]	​	​	χ^2^ = 1.736	0.629
*Aspergillus*	111 (54.7%)	29 (61.7%)	​	​
*Candida*	26 (12.8%)	7 (14.9%)	​	​
*Aspergillus* + *Candida*	8 (3.9%)	2 (4.3%)	​	​
Empirical use	58 (28.6%)	9 (19.1%)	​	​
Concomitant bacterial infection [*n* (%)]	96 (47.3%)	32 (68.1%)	χ^2^ = 6.605	0.010
Concomitant use of ≥3 antibacterial agents [*n* (%)]	19 (9.4%)	8 (17.0%)	χ^2^ = 2.326	0.127
Concomitant use of carbapenems [*n* (%)]	91 (44.8%)	27 (57.4%)	χ^2^ = 2.439	0.118
Concomitant use of polymyxin B [*n* (%)]	10 (4.9%)	4 (8.5%)	χ^2^ = 0.928	0.307
Concomitant use of linezolid [*n* (%)]	22 (10.8%)	9 (19.1%)	χ^2^ = 2.427	0.119
Concomitant use of piperacillin-tazobactam [*n* (%)]	48 (23.6%)	9 (19.1%)	χ^2^ = 0.438	0.508
Concomitant use of tigecycline [*n* (%)]	12 (5.9%)	6 (12.8%)	χ^2^ = 2.684	0.118
Concomitant use of quinolones [*n* (%)]	37 (18.2%)	6 (12.8%)	χ^2^ = 0.799	0.371
Concomitant use of ceftazidime [*n* (%)]	12 (5.9%)	2 (4.3%)	χ^2^ = 0.198	1.000
Concomitant use of trimethoprim-sulfamethoxazole [*n* (%)]	11 (5.4%)	3 (6.4%)	χ^2^ = 0.067	0.731
Concomitant use of proton pump inhibitor [*n* (%)]	143 (70.4%)	33 (70.2%)	χ^2^ = 0.001	0.975
Concomitant use of methylprednisolone [*n* (%)]	46 (22.7%)	5 (10.6%)	χ^2^ = 3.397	0.065
Concomitant use of dexamethasone [*n* (%)]	16 (7.9%)	4 (8.5%)	χ^2^ = 0.021	1.000
Loading dose [*n* (%)]	48 (23.6%)	12 (25.5%)	χ^2^ = 0.075	0.785
Administration [*n* (%)]	​	​	χ^2^ = 1.681	0.195
Oral	99 (48.8%)	18 (38.3%)	​	​
Intravenous	104 (51.2%)	29 (61.7%)	​	​
Duration of treatment [M(P_25_,P_75_)]	8.00 (6.00,13.00)	7.00 (5.00,15.00)	Z = −0.308	0.758
Dose/weight [mg/kg, M(P_25_,P_75_)]	6.15 (5.71,6.90)	6.67 (5.64,8.00)	Z = −1.644	0.100
C-_VRC_/dd [μg/L/mg, M(P_25_,P_75_)]	10.05 (7.04-14.29)	17.60 (11.31-21.21)	Z = −5.604	<0.001
C-_VRC_ [μg/mL, M(P_25_,P_75_)]	3.79 (2.61,5.31)	7.04 (4.53,8.49)	Z = −6.040	<0.001
C-_VNO_ [μg/mL, M(P_25_,P_75_)]	1.90 (1.47,2.43)	2.09 (1.05,2.57)	Z = −0.281	0.779
MR [M(P_25_,P_75_)]	1.99 (1.40,2.97)	3.52 (2.13,5.33)	Z = −4.720	<0.001
ALT [U/L, M(P_25_,P_75_)]	19.00 (11.70,27.70)	23.80 (14.00,33.30)	Z = −1.659	0.097
AST [U/L, M(P_25_,P_75_)]	23.30 (16.10,31.80)	30.40 (21.60,35.50)	Z = −1.878	0.060
GGT [U/L, M(P_25_,P_75_)]	38.70 (21.90,60.30)	46.00 (24.90,61.20)	Z = −0.697	0.486
ALP [U/L, M(P_25_,P_75_)]	80.00 (64.00,99.00)	81.00 (62.00,103.00)	Z = −0.264	0.792
TBIL [μmol/L, M(P_25_,P_75_)]	9.00 (6.80,13.80)	10.20 (6.60,17.00)	Z = −1.005	0.315
ALB [g/L, M(P_25_,P_75_)]	31.70 (28.35,35.45)	28.20 (25.95,31.45)	Z = −4.099	<0.001
CRP [mg/L,M(P_25_,P_75_)]	58.20 (17.60,109.30)	78.90 (36.90,129.50)	Z = −1.615	0.106
Scr [μmol/L, M(P_25_,P_75_)]	64.00 (52.00,82.00)	85.00 (57.00,124.00)	Z = −2.834	0.005

EMs: extensive metabolizers; IMs: intermediate metabolizers; PMs: poor metabolizers; COPD: chronic obstructive pulmonary disease; C-_VRC_: voriconazole steady-state trough concentration; DD: daily dose of voriconazole; C-_VNO_: voriconazole N-oxide concentration; MR: metabolic ratio, MR = C-_VRC_/C-_VNO_;ALT: alanine aminotransferase; AST: aspartate aminotransferase; GGT: gamma-glutamyl transferase; ALP: alkaline phosphatase; TBIL: total bilirubin; ALB: albumin; CRP: C-reactive protein; Scr: serum creatinine.

### LASSO regression and construction of the DILI nomogram prediction model

**FIGURE 2:**
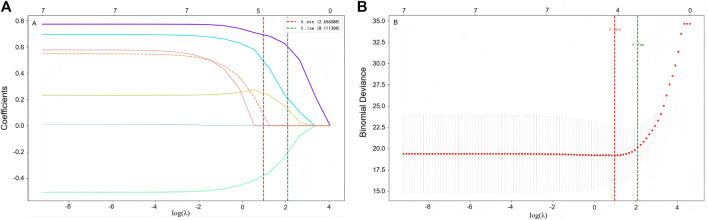
LASSO regression coefficient convergence path plot **(A)** and 10-fold cross-validation curve **(B)**.

The seven variables with statistically significant differences in univariate analysis were included in LASSO regression for further selection. As the penalty parameter λ increased, the regression coefficients of the variables gradually shrank toward zero. A 10-fold cross-validation curve for the LASSO regression was generated, with the left and right dashed lines representing λmin (2.656088) and λ1se (8.111308), respectively (Figure 2). Considering both model fit and predictive performance, λmin was selected as the optimal penalty parameter. Under this parameter, five predictors were ultimately identified as significantly associated with VRC-induced DILI: concomitant bacterial infection, C-_VRC_, MR, ALB and Scr.

**FIGURE 3:**
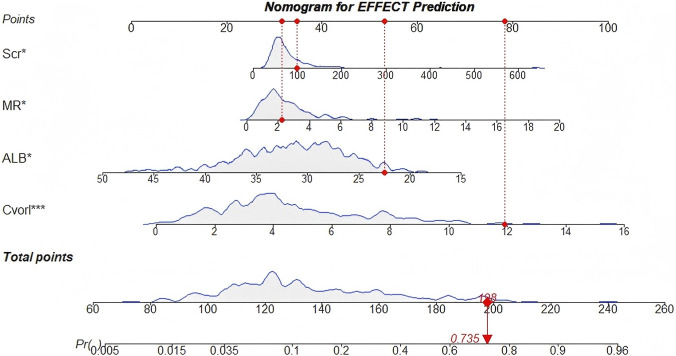
Logistic regression nomogram prediction model of voriconazole-induced DILI.

On the basis of the variables screened by LASSO regression, these predictors were entered into a multivariate logistic regression model using a stepwise selection method. The analysis identified C-_VRC_, MR, ALB, and Scr as independent factors associated with VRC-induced DILI (*P* < 0.05), as shown in Table 2. A nomogram for predicting the risk of VRC-induced DILI was subsequently constructed based on these predictors (Figure 3).

**TABLE 2:** Logistic regression of VRC-induced DILI.

Variables	β	SE	Wald	P	OR	95% CI	VIF
C-_VRC_	0.290	0.081	12.691	<0.001	1.336	1.139-1.567	1.47
MR	0.155	0.063	6.069	0.014	1.167	1.032-1.321	1.35
ALB	−0.102	0.041	6.169	0.013	0.903	0.834-0.979	1.09
Scr	0.004	0.002	5.340	0.021	1.004	1.001-1.008	1.02
Constant	−0.928	1.378	0.453	0.501	0.396	​	​

β: regression coefficient; SE: standard error; OR: odds ratio; CI: confidence interval; VIF: variance inflation factor.

### Evaluation and validation of the DILI nomogram prediction model

**FIGURE 4:**
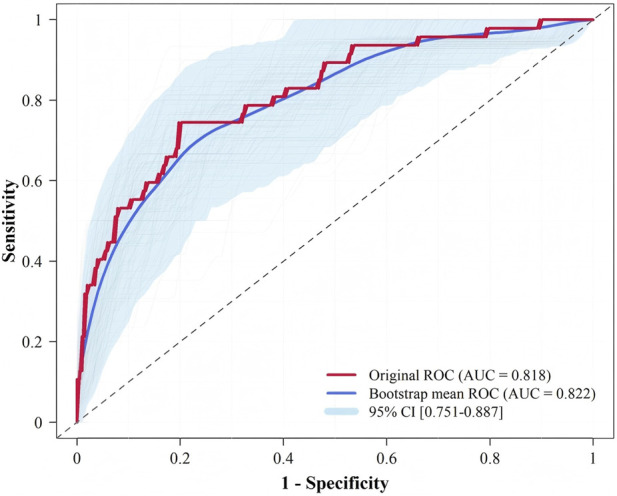
ROC curve of the voriconazole-induced DILI nomogram prediction model and the Boostrap method.

**FIGURE 5:**
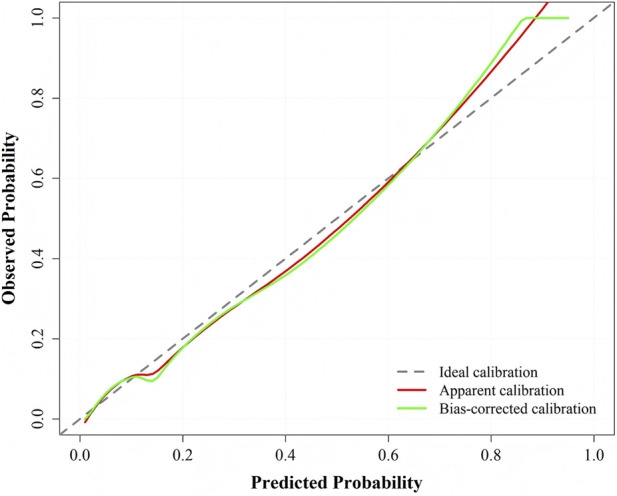
Calibration curve of voriconazole-induced DILI nomogram prediction model.

**FIGURE 6:**
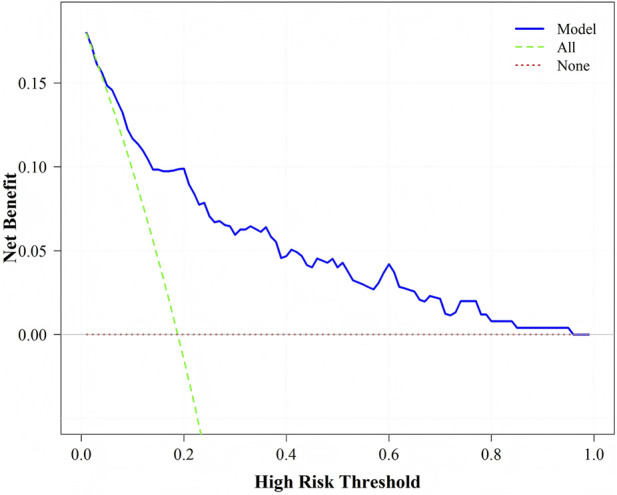
Decision curve of the voriconazole-induced DILI nomogram prediction model.

The receiver operating characteristic (ROC) curve was plotted to evaluate the discriminative performance of the nomogram. The area under the ROC curve (AUC) was 0.818 (95% CI: 0.748–0.888), indicating good discriminative ability. Bootstrap resampling with 1,000 iterations yielded an AUC of 0.822 (95% CI: 0.751–0.887) (Figure 4). Calibration was assessed using a calibration curve, and the Hosmer–Lemeshow test yielded a χ^2^ value of 10.178 (*P* = 0.2528), indicating good agreement between predicted and observed outcomes (Figure 5). Decision curve analysis (DCA) was used to evaluate the clinical utility of the model. The DCA curve demonstrated that across a threshold probability range of 4%–95%, the model provided a net clinical benefit for predicting DILI following VRC administration (Figure 6).

### Comparison of predictive performance among different variable combinations

To clarify the independent and combined predictive value of each indicator, single-factor models, two-variable combination models, three-variable combination models, and a four-variable full model were respectively constructed, and the performance of each model was systematically evaluated (Table 3). Based on the comparative analysis of model performance, the four-variable model (C-_VRC_ + MR + ALB + Scr) established in this study demonstrated the best predictive performance, with an AUC of 0.818, a sensitivity of 74.5%, and a specificity of 80.3% at an optimal threshold of 0.206. The three-variable combination models incorporating drug concentration also showed excellent performance, among which the C-_VRC_ + MR + ALB model achieved an AUC of 0.813, closely approaching that of the four-variable model. Univariate analysis revealed that C-_VRC_ had an AUC of 0.783, significantly superior to that of MR (0.721), indicating that drug concentration is the most important single predictor. In conclusion, the four-variable model exhibits the best comprehensive predictive performance, and the C-_VRC_ + MR + ALB combination can serve as a reliable alternative. The ROC curves are presented in the Supplementary Material.

**TABLE 3:** Performance comparison of different prediction models.

Model	AUC	Youden	Threshold	Sensitivity	Specificity
Four-variable	0.818	0.548	0.206	0.745	0.803
C-_VRC_ + MR+ALB	0.813	0.512	0.212	0.723	0.788
C-_VRC_ + MR + Scr	0.807	0.489	0.205	0.681	0.808
C-_VRC_ + ALB + Scr	0.804	0.451	0.191	0.702	0.749
C-_VRC_ + ALB	0.800	0.488	0.147	0.809	0.680
C-_VRC_ + MR	0.796	0.482	0.123	0.851	0.631
C-_VRC_ + Scr	0.794	0.450	0.324	0.553	0.897
C-_VRC_	0.783	0.442	0.111	0.915	0.527
MR + ALB + Scr	0.774	0.456	0.181	0.702	0.754
MR	0.721	0.392	0.186	0.575	0.818

### Development of a web-based predictive calculator

**FIGURE 7:**
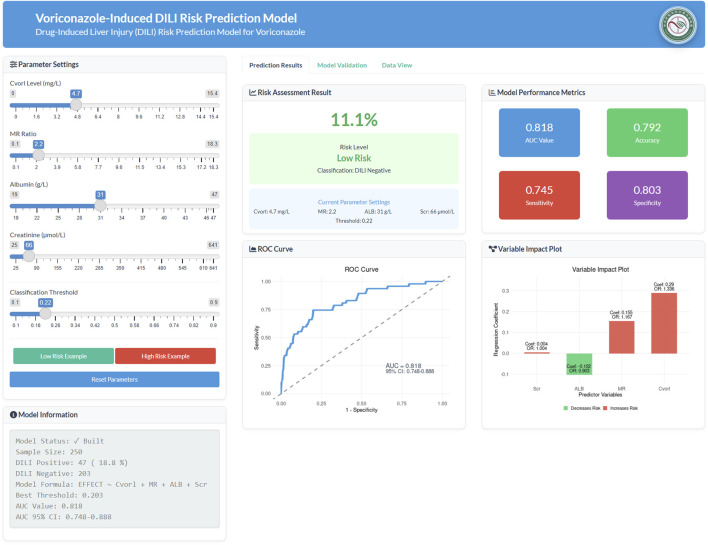
Web calculator for voriconazole-induced DILI risk prediction model.

To facilitate clinical application, a web-based interactive calculator was developed using R Shiny (https://dong1990.shinyapps.io/vorl_prediction/). By selecting the corresponding variable values in the parameter panel on the left side of the webpage, the predicted probability of VRC-induced DILI is automatically displayed on the result interface (Figure 7).

## Discussion

Our study is the first to construct a risk prediction model for VRC-induced DILI using the LASSO-logistic regression approach. Compared with traditional univariate analysis, LASSO offers significant advantages in handling multicollinearity, optimizing feature selection, and preventing model overfitting (Xi et al., 2023). The results showed that C_-VRC_, MR, ALB, and Scr were independent risk factors for VRC-induced DILI. The nomogram constructed based on these variables demonstrated good discrimination, calibration, and clinical utility. Bootstrap internal validation further confirmed the stability of the model. In addition, a web-based interactive prediction calculator was developed using R Shiny, providing a convenient tool for the prevention and management of liver injury risk associated with VRC use in clinical practice.

In recent years, a number of predictive models for VRC-induced DILI have been developed and validated (Du W. et al., 2025; Feng et al., 2025; Tong et al., 2024; Xiao et al., 2023). Xiao et al. (2023) constructed a logistic regression model based on hospitalized patients in China, the model validation results showed that the C-index of the derivation set and validation set was 0.706 and 0.733, respectively. Feng et al. (2025) developed a nomogram model using multivariable logistic regression analysis, which identified total cholesterol (TC) (OR = 1.893, P < 0.01), concomitant use of glucocorticoids (OR = 1.861, P = 0.041), ezetimibe (OR = 7.453, P = 0.047), and caspofungin (OR = 2.485, P = 0.032) as independent risk factors. The model achieved an AUC of 0.728. Du W. et al. (2025) focused on 97 lung transplant recipients and constructed a Cox regression-based predictive model incorporating voriconazole trough concentration, dosage, concomitant use of mycophenolate mofetil, and CYP2C19 genotype, achieving a C-index ranging from 0.807 to 0.820. Compared with previous studies, the present study may have some distinctive features. Notably, the MR was incorporated into the prediction model, which might complement the limitation of relying solely on trough concentrations. Additionally, LASSO regression was employed for variable selection, which might help avoid the potential overfitting associated with traditional univariate screening approaches. Moreover, a web-based interactive prediction calculator was developed to enable real-time DILI risk assessment, which might enhance clinical usability compared with models that are provided only as nomograms.

The occurrence of VRC-related DILI is influenced by multiple factors, including plasma concentration, genetic predisposition, and underlying diseases. Among these, elevated VRC plasma concentration serves as a direct and critical independent risk factor for DILI, particularly when the trough concentration persistently exceeds 4–5 mg/L, at which point the risk increases markedly (Lin et al., 2023; Suzuki et al., 2013; Taghvaye-Masoumi et al., 2021). These findings are consistent with those of the present study, in which 87.2% (91/203) of patients in the DILI group had VRC plasma concentrations >4 μg/mL, which was significantly higher than the 44.8% (91/203) observed in the non-DILI group. Moreover, according to the LASSO regression results, VRC plasma concentration had a substantial contribution to the model, suggesting that therapeutic drug monitoring of VRC is an important strategy for preventing and managing the risk of DILI in clinical practice. Furthermore, this study extended beyond the single indicator of VRC plasma concentration by incorporating the concentration of the major metabolite VNO and MR. The results indicated that an elevated MR was positively associated with the risk of liver injury. VNO is the primary metabolite of VRC in the liver via CYP2C19 metabolism. Cheng et al. (2024) demonstrated using ROC curve analysis that when the VRC plasma concentration exceeded 4.0 μg/mL or the VNO plasma concentration fell below 1.7 μg/mL, the incidence of grade 1 or higher adverse events related to aspartate aminotransferase and total bilirubin increased significantly. Wang H. et al. (2024) analyzed differences in VRC and VNO concentrations and the C_-VNO_/C_-VRC_ ratio in patients undergoing stem cell transplantation, and found that the C_-VNO_/C_-VRC_ ratio was significantly lower in the liver injury group compared with the normal group, which is consistent with the findings of the present study. MR serves as a direct indicator of the metabolic capacity for VRC conversion into VNO. An increase in the MR value suggests impaired drug metabolism, resulting in ssystemic drug accumulation and an elevated risk DILI. Consequently, relying solely on VRC plasma concentration monitoring may not adequately assess the full spectrum of liver injury risk. Integrated monitoring of VNO levels and the MR can offer a more precise representation of a patient’s drug exposure and metabolic profile, thus providing a more holistic foundation for risk assessment.

Comparative analysis of model performance indicated that C_-VRC_ was the most important predictor among the four variables. In single-factor models, C_-VRC_ showed a significantly higher AUC (0.783) than MR (0.721). Among combination models, those incorporating C_-VRC_ consistently demonstrated substantially higher AUC values than those without C_-VRC_. For instance, the C_-VRC_ + MR + ALB model yielded an AUC of 0.813, whereas the MR + ALB + Scr model achieved only 0.774. These findings further confirm that C_-VRC_ is the most critical predictor in our model. To investigate the potential collinearity between C_-VRC_ and MR, variance inflation factor (VIF) analysis was performed in this study. The results showed that the VIF values for C_-VRC_ and MR were 1.47 and 1.35, respectively, both well below the threshold of 5, indicating no significant collinearity between the two variables. Pearson correlation analysis revealed no strong correlation between them (r = 0.51). However, comparative analysis of model performance revealed that the improvement in AUC after adding MR was modest. Specifically, the addition of MR to the C_-VRC_ + ALB model increased the AUC from 0.800 to 0.813, while its addition to the C_-VRC_ + Scr model increased the AUC from 0.794 to 0.807. These findings suggest that the incremental contribution of MR to the overall predictive performance of the model is relatively limited. Nevertheless, as an important indicator reflecting VRC metabolic capacity, the inclusion of MR remains mechanistically meaningful. A recent study demonstrated that VNO may induce hepatocyte injury through oxidative stress (Du Q. et al., 2025). An elevated MR may indicates impaired VRC metabolism and consequent drug accumulation, thereby increasing the risk of liver injury.

Furthermore, the findings of this study revealed a significant association between both Scr and ALB levels and the risk of VRC-induced DILI. This observation aligns consistently with previous research (Liu et al., 2022; Xiao et al., 2023). Although voriconazole is primarily metabolized by the liver with negligible renal excretion (Leveque et al., 2006), recent population pharmacokinetic studies in critically ill patients have identified creatinine clearance as a significant covariate affecting voriconazole clearance (Wang Y. et al., 2024; Zhang et al., 2026). A previous study reported higher voriconazole plasma concentrations in patients with moderate renal impairment than in those with normal renal function following the administration of 320 mg and 240 mg doses (Myrianthefs et al., 2010). In a population pharmacokinetic study conducted among renal transplant recipients, the population clearance of voriconazole was determined to be 2.88 L/h, which was lower than the data obtained from other patients with invasive fungal infections; the authors suggested that this reduced clearance might be attributed to incomplete recovery of renal function in these recipients (Lin et al., 2018). Therefore, despite the discrepancy between theory and clinical observations, Scr was identified as an independent predictor of VRC-induced DILI in our study (OR = 1.004, P = 0.021). This association may reflect the role of creatinine as a surrogate marker for disease severity and overall physiological status, rather than a direct effect on voriconazole clearance.

Approximately 50% of voriconazole binds to plasma proteins, primarily albumin. Clinical studies have demonstrated that hypoalbuminemia is associated with increased unbound voriconazole concentrations (Florent et al., 2014; Vanstraelen et al., 2014). Vanstraelen et al. found a significant positive correlation between albumin levels and protein binding (Vanstraelen et al., 2014). However, Florent et al. reported no correlation between serum albumin levels and unbound fraction, except for some patients with severe hypoalbuminemia (Florent et al., 2014). In our study, the DILI group had significantly lower albumin levels (median 28.20 vs. 31.70 g/L, P < 0.001), and the proportion of patients with severe hypoalbuminemia (ALB < 25 g/L) was 2.8 times higher in the DILI group (19.1%) than in the non-DILI group (6.9%, *P* = 0.019). Furthermore, a relevant clinical study (Li et al., 2025) demonstrated a significant positive correlation between VRC trough concentrations and liver injury markers such as AST and ALP levels in hypoalbuminemic patients, with elevated trough concentrations identified as an independent risk factor for hepatotoxicity. This suggests that in clinical therapeutic drug monitoring practice, patients with concomitant hypoalbuminemia warrant more vigilant monitoring of liver function and free drug concentrations, and consideration should be given to adopting more conservative upper limits for trough concentration targets. Therefore, hypoalbuminemia may increase the risk of voriconazole-induced liver injury by affecting unbound voriconazole concentrations and reflecting disease severity. However, future prospective studies and *in vitro* pharmacokinetic investigations are warranted to clarify the effect of hypoalbuminemia in voriconazole-induced hepatotoxicity.

Compared to previous clinical predictive model studies, this research developed a web-based interactive calculator using R-shiny, deploying a dynamic predictive model for VRC-induced DILI. Unlike traditional nomogram models, clinicians can input corresponding parameters to instantly calculate the probability of DILI occurrence, offering ease of operation, high visualization, and close alignment with clinical needs. However, this study still possesses certain limitations: First, the sample size is relatively modest, which may limit the statistical power and stability of the model. Second, the research data were derived from single-center retrospective data, and despite the model’s robust internal validation performance, its population representativeness is limited. The model’s generalizability and external validity therefore urgently require further validation through multi-center, large-sample prospective studies. Additionally, populations such as long-term medication users and outpatients were not included, thus the model’s applicability in different clinical scenarios awaits further verification. Furthermore, although this study analyzed CYP2C19 gene polymorphism, this indicator was not incorporated into the final model, which may be attributed to sample size and ethnic differences. Future multi-center, large-sample prospective studies are warranted to externally validate and refine the proposed prediction model, as well as to further investigate the association between genetic factors and DILI.

## Data Availability

The raw data supporting the conclusions of this article will be made available by the authors, without undue reservation.

## Supplementary material

SUPPLEMENTARY FIGURE S1 ROC curves of the four-variable model, best single-factor model, and best combination model for predicting voriconazole-induced DILI.

SUPPLEMENTARY FIGURE S2 AUC ranking of the ten prediction models. The four-variable model (red bar) achieved the highest AUC among all models.

## References

[B1] Cheng L. You X. Wang X. Yu M. Jia C. (2024). The role of plasma trough concentration of voriconazole and voriconazole N-Oxide in its hepatotoxicity in adult patients. Drug Des. Devel Ther. 18, 3617–3628. 10.2147/DDDT.S475706 39156484 PMC11330242

[B2] Du Q. Qiu Y. Yang L. Wang C. Teng M. Chen J. (2025). Mechanism and marker of voriconazole-induced liver injury: insights from a quantitative systems toxicology approach. Regul. Toxicol. Pharm. 162, 105871. 10.1016/j.yrtph.2025.105871 40447069

[B3] Du W. Chen W. Zhang D. Li S. Li B. Zuo X. (2025). Establishment and validation of a predictive nomogram for voriconazole-associated liver injury in lung transplant patients. Int. J. Clin. Pharm-Net 47, 1416–1426. 10.1007/s11096-025-01946-8 40668536

[B4] Eiden C. Peyriere H. Cociglio M. Djezzar S. Hansel S. Blayac J. (2007). Adverse effects of voriconazole: analysis of the French pharmacovigilance database. Ann. Pharmacother. 41, 755–763. 10.1345/aph.1H671 17456542

[B5] Feng D. Ma Y. Liu X. Tang S. Xiang S. Wang J. (2025). Voriconazole-associated liver injury: clinical risk factor identification and predictive nomogram construction. Front. Pharmacol. 16, 1688711. 10.3389/fphar.2025.1688711 41560757 PMC12813146

[B6] Florent A. Gandia P. Seraissol P. Chatelut E. Houin G. (2014). Determination of plasma unbound fraction of voriconazole in patients treated with a prophylactic or a curative treatment. Ther. Drug Monit. 36, 752–758. 10.1097/FTD.0000000000000095 24819971

[B7] Leveque D. Nivoix Y. Jehl F. Herbrecht R. (2006). Clinical pharmacokinetics of voriconazole. Int. J. Antimicrob. Ag. 27, 274–284. 10.1016/j.ijantimicag.2006.01.003 16563707

[B8] Li Y. Xu H. Wang H. Zhang W. Song Y. Wang S. (2025). Study on the correlation between plasma concentration of voriconazole and clinical efficacy and safety in elderly patients. Front. Pharmacol. 16, 1726902. 10.3389/fphar.2025.1726902 41560750 PMC12813152

[B9] Lin X. Li Z. Yan M. Zhang B. Liang W. Wang F. (2018). Population pharmacokinetics of voriconazole and CYP2C19 polymorphisms for optimizing dosing regimens in renal transplant recipients. Brit. J. Clin. Pharm. 84, 1587–1597. 10.1111/bcp.13595 29607533 PMC6005582

[B10] Lin L. Hong M. Wu D. Zhong L. Fu X. (2023). The status of therapeutic drug monitoring and voriconazole-induced liver injury and its influencing factors. Chin. J. Infect. Chemother. 06, 709–714. 10.16718/j.1009-7708.2023.06.007

[B11] Liu L. Yang W. Peng J. Hu Z. (2022). Construction and validation of a risk prediction model for voriconazole-induced liver injury. J. Central South Pharm. 20, 2906–2910.

[B12] Lou Y. Wang Y. Liu J. Wang Y. Wang J. Ma S. (2025). Voriconazole-induced liver injury: incidence patterns and risk factors in a retrospective cohort. Antimicrob. Agents Ch. 69, e48725. 10.1128/aac.00487-25 40741951 PMC12406674

[B13] Mao Y. M. (2023). Standardize the diagnosis and treatment of drug-induced liver injury, and strengthen clinical and translational research. Zhonghua Gan Zang Bing Za Zhi 31, 337–338. 10.3760/cma.j.cn501113-20230419-00176 37248972 PMC12854814

[B14] Matsumoto K. Ikawa K. Abematsu K. Fukunaga N. Nishida K. Fukamizu T. (2009). Correlation between voriconazole trough plasma concentration and hepatotoxicity in patients with different CYP2C19 genotypes. Int. J. Antimicrob. Ag. 34, 91–94. 10.1016/j.ijantimicag.2009.01.008 19261446

[B15] Myrianthefs P. Markantonis S. L. Evaggelopoulou P. Despotelis S. Evodia E. Panidis D. (2010). Monitoring plasma voriconazole levels following intravenous administration in critically ill patients: an observational study. Int. J. Antimicrob. Ag. 35, 468–472. 10.1016/j.ijantimicag.2009.12.021 20188523

[B16] Patterson T. F. Thompson G. R. R. Denning D. W. Fishman J. A. Hadley S. Herbrecht R. (2016). Practice guidelines for the diagnosis and management of aspergillosis: 2016 update by the infectious diseases society of America. Clin. Infect. Dis. 63, e1–e60. 10.1093/cid/ciw326 27365388 PMC4967602

[B17] Rakhshan A. Rahmati Kamel B. Saffaei A. Tavakoli-Ardakani M. (2023). Hepatotoxicity induced by azole antifungal agents: a review Study. Iran. J. Pharm. Res. 22, e130336. 10.5812/ijpr-130336 38116543 PMC10728840

[B18] Ren J. Cai X. Ge W. Guo J. Wang S. Wang Q. (2025). Risk factors for voriconazole-associated hepatotoxicity in patients with liver dysfunction: a retrospective nested case-control study. Front. Pharmacol. 16, 1625003. 10.3389/fphar.2025.1625003 40932865 PMC12417474

[B19] Suzuki Y. Tokimatsu I. Sato Y. Kawasaki K. Sato Y. Goto T. (2013). Association of sustained high plasma trough concentration of voriconazole with the incidence of hepatotoxicity. Clin. Chim. Acta 424, 119–122. 10.1016/j.cca.2013.05.025 23747486

[B20] Taghvaye-Masoumi H. Hadjibabaie M. Ghadimi M. Zarif-Yeganeh M. Vaezi M. Ghavamzadeh A. (2021). Association of Voriconazole trough plasma concentration with efficacy and incidence of hepatotoxicity in Iranian patients with hematological malignancies. Iran. J. Pharm. Res. 20, 62–71. 10.22037/ijpr.2020.112330.13688 34400941 PMC8170753

[B21] Tang D. Yan M. Song B. Zhao Y. Xiao Y. Wang F. (2021). Population pharmacokinetics, safety and dosing optimization of voriconazole in patients with liver dysfunction: a prospective observational study. Brit. J. Clin. Pharm. 87, 1890–1902. 10.1111/bcp.14578 33010043

[B22] Tong D. Wang Y. Ma J. Wang J. Li J. (2024). Construction of a prediction model for voriconazole-induced hepatotoxicity based on mixed-effects random Forest. Stud. Health Technol. Inf. 310, 319–323. 10.3233/SHTI230979 38269817

[B23] Vanstraelen K. Wauters J. Vercammen I. de Loor H. Maertens J. Lagrou K. (2014). Impact of hypoalbuminemia on voriconazole pharmacokinetics in critically ill adult patients. Antimicrob. Agents Ch. 58, 6782–6789. 10.1128/AAC.03641-14 25182655 PMC4249353

[B24] Wang Y. Wang T. Xie J. Yang Q. Zheng X. Dong W. (2016). Risk factors for voriconazole-associated hepatotoxicity in patients in the intensive care unit. Pharmacotherapy 36, 757–765. 10.1002/phar.1779 27284960

[B25] Wang H. Wang L. Li M. Shi L. Sun H. Liu H. (2024). The application study of voriconazole and its metabolites concentration monitoring in allogeneic hematopoietic stem cell transplantation patients. 32, 945–951. 10.19746/j.cnki.issn.1009-2137.2024.03.045 38926993

[B26] Wang Y. Ye Q. Li P. Huang L. Qi Z. Chen W. (2024). Renal replacement therapy as a new indicator of voriconazole clearance in a population pharmacokinetic analysis of critically ill patients. Pharmaceuticals-Base 17, 665. 10.3390/ph17060665 38931333 PMC11206427

[B27] Xi L. J. Guo Z. Y. Yang X. K. Ping Z. G. (2023). Application of LASSO and its extended method in variable selection of regression analysis. Zhonghua Yu Fang. Yi Xue Za Zhi 57, 107–111. 10.3760/cma.j.cn112150-20220117-00063 36655266

[B28] Xiao G. Liu Y. Chen Y. He Z. Wen Y. Hu M. (2023). The development and validation of a predictive model for voriconazole-related liver injury in hospitalized patients in China. J. Clin. Med. 12, 4254. 10.3390/jcm12134254 37445289 PMC10342760

[B29] Xiao Z. Zhang X. Zhang L. Li Y. Ma S. (2025). Drug-induced liver injury highly probable due to goserelin: a case report evaluated with the updated RUCAM (2016). Front. Med-Lausanne 12, 1683370. 10.3389/fmed.2025.1683370 41416092 PMC12708907

[B30] Zhang Y. Hou K. Liu F. Luo X. He S. Hu L. (2021). The influence of CYP2C19 polymorphisms on voriconazole trough concentrations: systematic review and meta-analysis. Mycoses 64, 860–873. 10.1111/myc.13293 33896064

[B31] Zhang D. Du W. Qin W. Li B. Li P. Wang X. (2026). Population pharmacokinetic and pharmacodynamic analysis of voriconazole‐induced liver injury in Chinese lung transplant patients. J. Clin. Pharm. Ther.,2026, 4521997. 10.1155/JCPT/4521997

[B32] Zhou Z. Yin X. Zhang Y. Shao Q. Mao X. Hu W. (2022). Antifungal drugs and drug-induced liver injury: a real-world study leveraging the FDA adverse event reporting system database. Front. Pharmacol. 13, 891336. 10.3389/fphar.2022.891336 35571077 PMC9098189

