# Supplementary material for: Incorporating the metabolic ratio (C-VRC/C-VNO) into a LASSO-logistic model for predicting voriconazole-induced DILI: development and web-based implementation
Source: Front Pharmacol. 2026 Jul 6;17:1857552. doi: 10.3389/fphar.2026.1857552 (PMC13382107; doi:10.3389/fphar.2026.1857552)
Supplement: Supplementary file 1 [file Supplementaryfile1.docx]

Supplementary materials


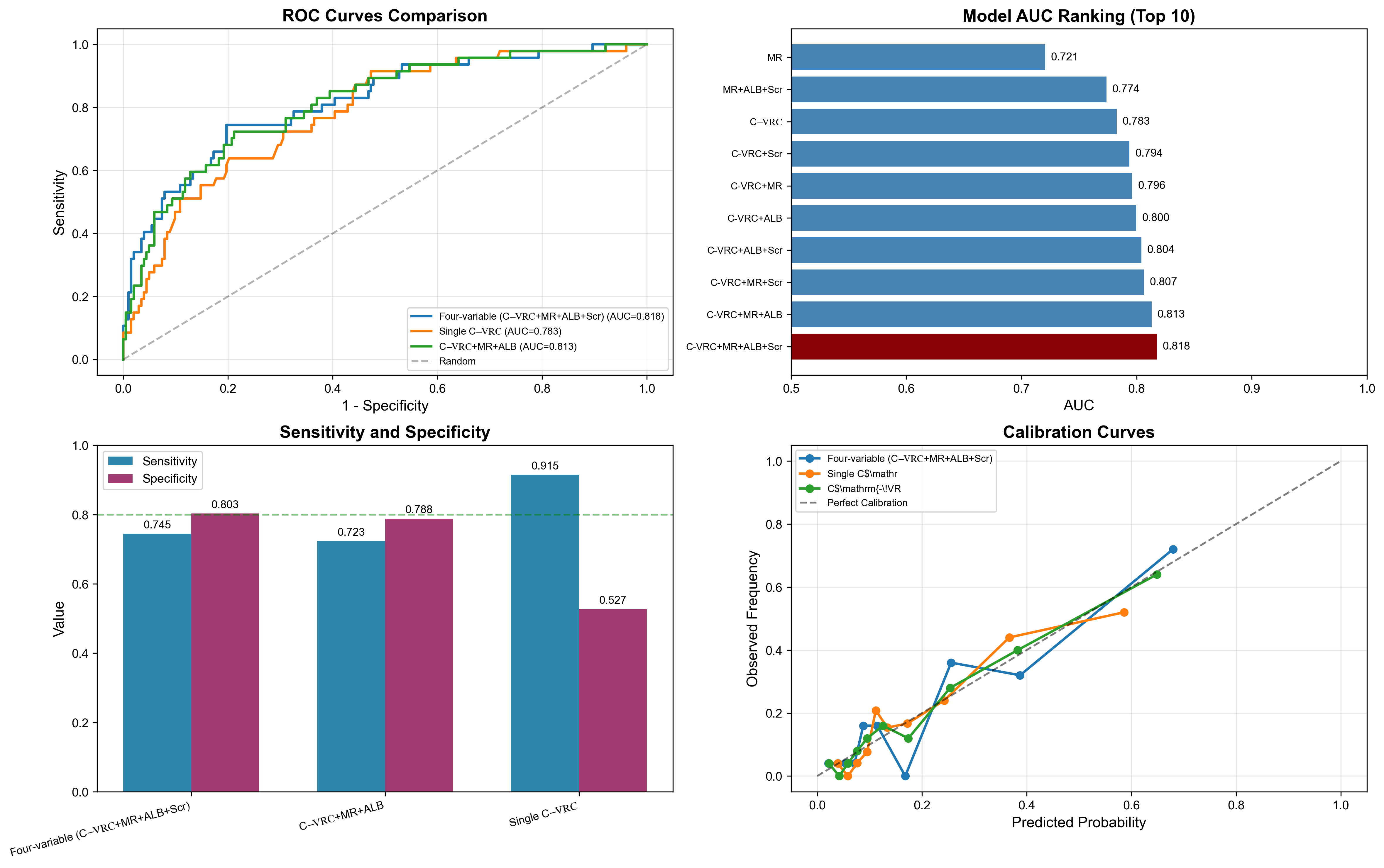


Supplementary Figure 1 ROC curves of the four-variable model, best single-factor model, and best combination model for predicting voriconazole-induced DILI


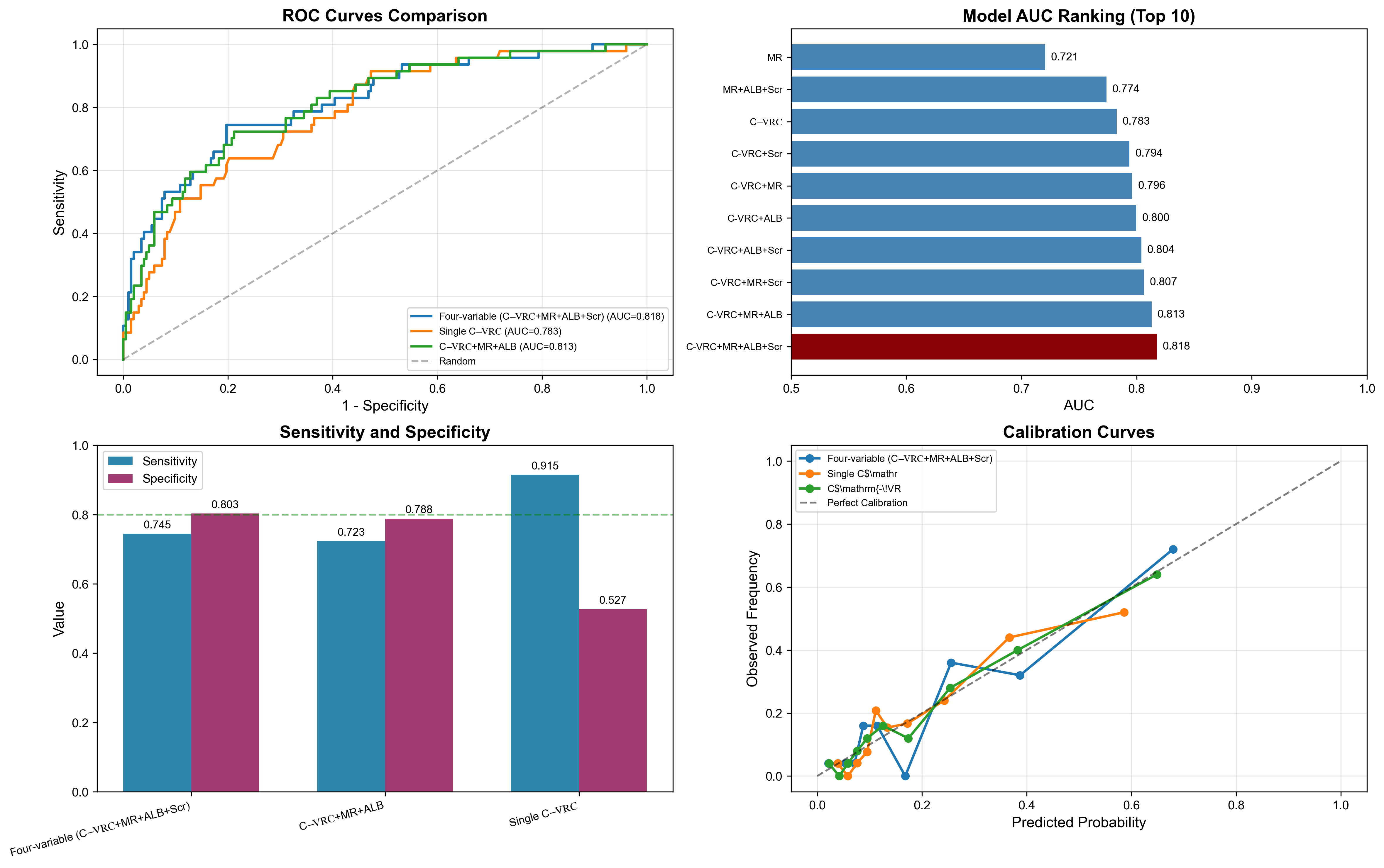


Supplementary Figure 2 AUC ranking of the ten prediction models. The four-variable model (red bar) achieved the highest AUC among all models.
